# Thermal effect of holmium laser during ureteroscopic lithotripsy

**DOI:** 10.1186/s12894-020-00639-w

**Published:** 2020-06-15

**Authors:** Hui Liang, Lijian Liang, Yin Yu, Bin Huang, Jia’nan Chen, Chaoguo Wang, Zhangguo Zhu, Xiaozhong Liang

**Affiliations:** Department of Urology, Xinchang County Hospital of Traditional Chinese Medicine, Shaoxing, Zhejiang Province China

**Keywords:** Holmium laser, Thermal effect, Lithotripsy

## Abstract

**Background:**

Holmium laser lithotripsy is the most common technique for the management of ureteral stone. Studies founded that holmium laser firing can produce heat which will cause thermal injury towards ureter. The aim of our current study is to explore factors affecting thermal effect of holmium laser during ureteroscopic lithotripsy.

**Methods:**

An in vitro experimental model is design to simulate the ureteroscopic lithotripsy procedure. Different laser power settings (10w (0.5JX20Hz, 1.0 JX10Hz), 20w (1.0 JX20Hz, 2.0 JX10Hz), 30w (1.5JX20Hz, 3.0 JX10Hz)) with various firing time (3 s, 5 s, 10s) and irrigation flow rates(10 ml/min, 15 ml/min, 20 ml/min and 30 ml/min) were employed in the experiment. The temperature around the laser tip was recorded by thermometer.

**Results:**

The temperature in the “ureter” rises significantly with the increasing laser power, prolonging firing time and reducing irrigation flow. The highest regional temperature is 78.0 °C at the experimental set-up, and the lowest temperature is 23.5 °C. Higher frequency setting produces more heat at the same power. Laser power < =10w, irrigation flow> = 30 ml/min and “high-energy with low-frequency” can permit a safe working temperature.

**Conclusion:**

We clarify that the thermal effect of holmium laser is related with both laser working parameters and irrigation flow. The proper setting is the key factor to ensure the safety during ureteroscopic holmium laser lithotripsy.

## Background

Ureteral calculus is a common urological entity which is usually managed by ureteroscopic lithotripsy. The Holmium:YTTRIUM-ALUMINUM-GARNET (Ho:YAG) laser is the most efficient and versatile modality for ureteroscopic management of ureteral calculus [1]. Holmium laser’s safety profile has been previously shown to be favorable compared to other lithotrites, since the 2100-nm wavelength of the Ho:YAG laser permits rapid absorption in water and thereby limits the depth of penetration and surrounding tissue injury. However, with the widely application of Ho:YAG laser, complications entailed by ureteroscopy such as ureteral stricture become common. It is reported that post-ureteroscopy ureteral stricture can be as high as 1–4% [2]. The potential cause of stricture is more likely to be the direct thermal injury towards the ureteral wall rather than acoustic or photonic energy [3]. Laser energy can produce heat and potentially cause thermal bioeffects [4–7]. In our current study, we aim to characterize the relationship between working parameter and intra-ureteral temperatures during ureteroscopic holmium lithotripsy.

## Methods

**Fig. 1:**
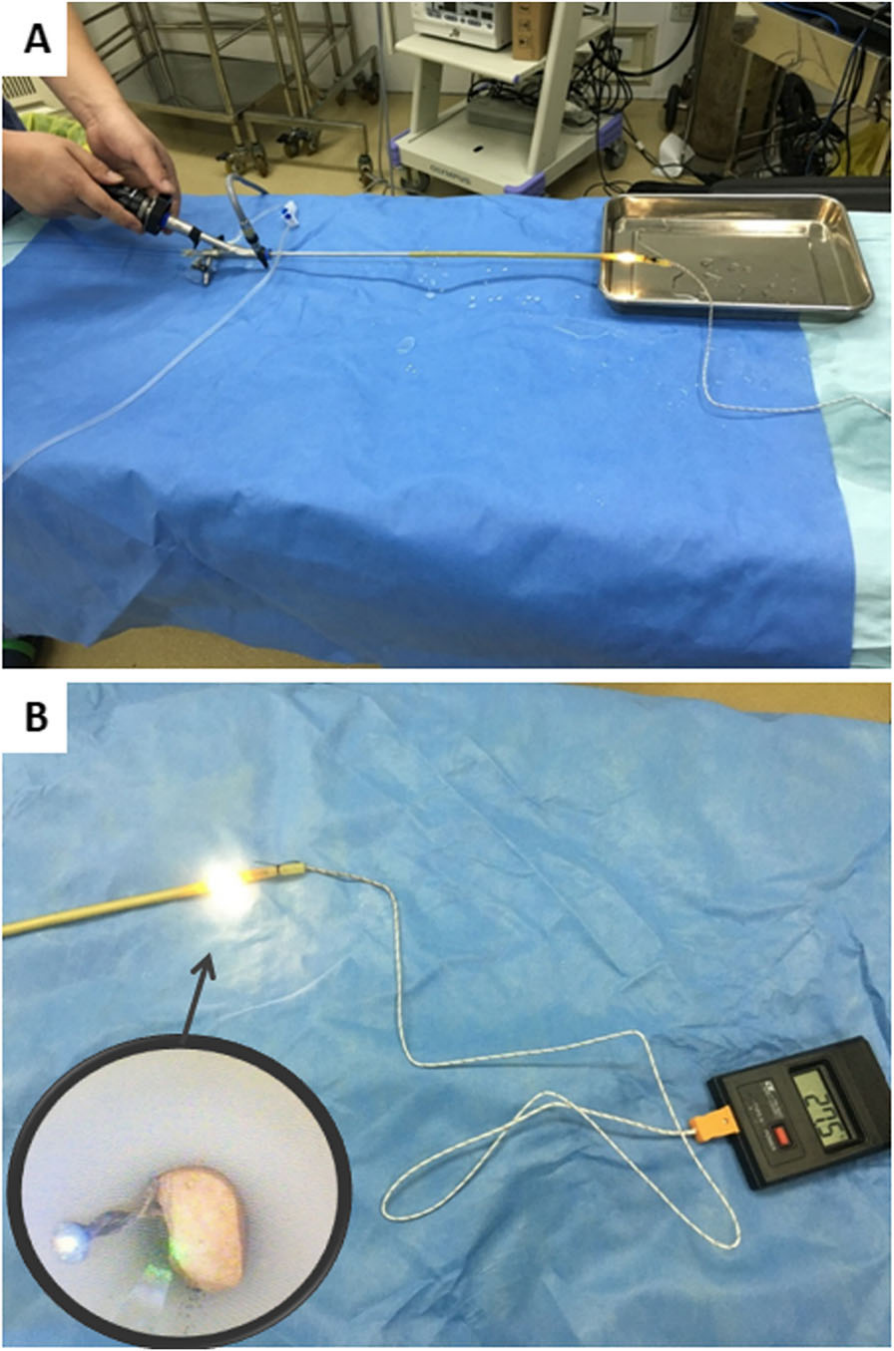
**a** A simulated ureteroscopic laser lithotripsy procedure was established within a rubber tube. **b** Regional temperature was recorded by thermocouple

An in vitro model mimicking ureteroscopic laser lithotripsy was established to assess the temperature changes during laser discharge. A segment of ligated F20 rubber tube was used to simulating human ureter with stone impaction. The lithotripsy laser system was used with the 200um laser fiber passed through the working channel of a F8/9.8 ureteroscope (Fig. 1). Raykeen Holmium laser (Raykeen, Shanghai, China) was applied in the experiment with the various power settings commonly used in clinical practice. Energy settings were 0.5, 1.0, 1.5 and 2.0 J. Frequency settings of 10 and 20 Hz were tested at different energy settings. Laser firing time was 3 s, 5 s and 10s. The relevant irrigation flow rates of 10 ml/min, 15 ml/min, 20 ml/min and 30 ml/min were employed by infusion pump at room temperature. A thermocouple was held 5 mm from the bottom and approximately 2 mm lateral from the position of the laser tip to record temperature. The temperature for each power and irrigation setting combination was recorded five times, and means were calculated for statistical comparison in different setup.

## Results

Heat accumulated around the laser fiber tip. The temperature data was summarized in Table 1. The temperature rises with the increasing laser power and firing time. Adequate irrigation flow can carry the heat away. The peak temperature, 78.0 °C, occurred with continuous laser firing for 10s at the power setting of 1.5JX20Hz and irrigation flow rate 10 ml/min. While the lowest temperature, 25.3 °C, was captured after continuous laser firing for 3 s at the power setting of 1.0 JX10Hz and irrigation flow rate 30 ml/min.

**Table 1:** Regional temperature changes with different laser working models and irrigation flow rates

Power	Flow rates	10 ml/min			15 ml/min			20 ml/min			30 ml/min		
	Firing Time	3 s	5 s	10s	3 s	5 s	10s	3 s	5 s	10s	3 s	5 s	10s
10w	0.5 J,20HZ	32.0	34.9	34.3	29.6	28.4	32.0	29.7	30.8	31.4	28.2	28.0	28.7
	1.0 J,10HZ	30.6	31.9	30.8	29.0	29.6	30.3	28.8	28.6	29.8	25.3	26.1	26.4
20w	1.0 J,20HZ	56.3	65.2	73.5	42.2	54.6	56.2	37.9	39.4	39.5	35.4	36.5	36.1
	2.0 J,10HZ	45.3	47.3	55.0	36.5	40.4	48.9	34.0	34.3	35.5	31.8	31.4	32.1
30w	1.5 J,20HZ	63.1	67.3	78.0	55.6	58.2	59.9	44.7	48.2	52.3	34.5	37.4	38.8
	3.0 J,10HZ	55.8	57.5	55.5	39.7	47.9	51.8	37.5	40.5	41.9	30.7	32.9	34.9

**Fig. 2:**
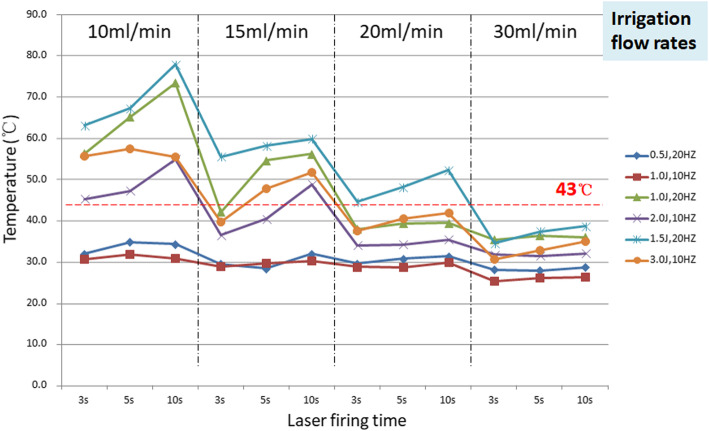
Temperature curve of different laser power setting

For the power setting of 10w, the temperature remains more stable irrespective of laser firing time or irrigation flow rate. When the power reaches 20w, the temperatures rapidly rise if irrigation is insufficient. Adequate irrigation or shorter laser firing time will be required to restrict the temperature. Figure 2 depicts temperature tracings for trials with different laser settings at 4 different irrigation flow rates. Another interesting finding is that for the same power setting, the “high-energy with low-frequency” parameter generates less thermal effect. The mean temperature difference is more evident when the irrigation is insufficient.

## Discussion

Holmium laser is the most powerful energy tool for stone fragmentation during ureteroscopic lithotripsy [8]. Even though it is regarded as a minimally invasive surgical approach, it still brings troublesome complication, such as ureteral stricture [9]. Ureteral stricture is one of common complication post ureteroscopic surgery, especially for the impacted ureteral stone [9]. Previously, mechanical injury is considered as the main cause for ureteral stricture. However, more and more evidence indicates ureteral stricture can also occur even without mechanical injury. The issue of the intra-opertative holmium laser thermal effect has gradually gained attention [3, 10]. Thermal effect stemmed from holmium laser is considered the hidden etiology for ureteral stricture.

The holmium laser is a long-wavelength pulsed laser, which can transform the energy into optomechanical/photoacoustic and photothermal effect during calculi fragmentation [11, 12]. The estimated mechanical effect to turn a stone into fragments is small. Most of energy will turn into the thermal effect boiling the fluid around the laser tip. Numerous studies have confirmed that significant temperature rise can occur around the fiber. Once, the temperature exceeds the “threshold” (43 °C), it can lead to cell damage, protein coagulation and tissue injury [13], which subsequentially progresses to scar formation and ureteral stricture. According to the relationship of the tissue thermal damage and temperature, for every 1 °C exceeds the threshold temperature, the time required to cause damage to the same number of cells will decrease by half [14]. Therefore, when the temperature reaches 56 °C, it takes only 1 s to cause thermal damage to the tissue. So, the thermal injury towards ureter will be easily neglected during lithotripsy procedure.

In our current study, we established an in vitro model to evaluate the thermal effect of holmium laser and the protection effect of fluid irrigation during ureteroscopic laser lithotripsy. According to our model, the temperature rise causing by laser firing depends on the laser power, working time and fluid irrigation. Higher power laser can increase temperature much more quickly. The 20w laser setting can increase the temperature as high as 73.5 °C if irrigation is insufficient. Meanwhile, the high-frequency dusting model of holmium laser is much more efficient in heat production. Fluid irrigation plays an important role in regional heat dispersion. The flow rate up to 20 ml/min seems to be the optimal requirement during the laser lithotripsy while considering the intra-renal pelvis pressure and stone retropulsion at the same time. When the flow rate reaches 30 ml/min, the temperature can not surpass the threshold.

Thus, we provide several feasible suggestions to prevent thermal injury during endoscopic laser lithotripsy, especially for impacted ureteral calculi. First, lithotripsy is advised start with low power setting. Generally, low power setting is sufficient to fragment majority of calculi. We recommend that the power setting should not exceed 20w for most devices. Second, apply “high-frequency” setting with more caution. Previous study have showed that dusting maneuver with higher frequency can acquire better stone free rates for renal calculi [15]. Even though the dusting maneuver is more time-consuming and will produce more heat, the renal pelvis can disperse the heat quickly because of its larger space comparing with ureter. So, try to avoid the “high-frequency” laser parameter for stone dusting in the ureter. Third, ensure the fluid irrigation during the procedure. Irrigation flow should be sufficient to clear field of view and cool down the regional temperature. Using a smaller caliber ureteroscope accompanied with fine laser fiber can facilitate a better fluid irrigation and counter-flow. Last, for the impacted calculi, in-situ lithotripsy will be clumsy [9]. It can impair the fluid irrigation by blocking fluid circulation and cause the direct injury towards the ureter mucosa at the impacted site. So, we suggest displace the calculi to proximal portion of ureter prior to calculi fragmentation, since the dilated ureter will provide much more space for fluid irrigation and have less chance of direct injury by the laser fiber.

## Conclusion

In conclusion, we found that laser heating can cause significant temperature rise in the ureter with various laser parameters and duration of pulsing. Laser can cause irreversible thermal injury towards ureter. Thus, during clinical application of laser lithotripsy technique, caution should be taken.

## Data Availability

The datasets used and analysed during the current study are available from the corresponding author on reasonable request. All authors have read the paper and agree that it can be published.

## Abbreviations

Holmium: YTTRIUM-ALUMINUM-GARNET
Ho: YAG
